# Prevalence of and factors associated with swellings of the ribs in tie stall housed dairy cows in Germany

**DOI:** 10.1371/journal.pone.0269726

**Published:** 2022-07-15

**Authors:** Greta E. Abele, Yury Zablotski, Melanie Feist, K. Charlotte Jensen, Annegret Stock, Amely Campe, Roswitha Merle, Andreas W. Oehm

**Affiliations:** 1 Clinic for Ruminants with Ambulatory and Herd Health Services, Ludwig-Maximilians Universität Munich, Oberschleissheim, Germany; 2 Clinic for Cattle, University of Veterinary Medicine, Foundation, Hannover, Germany; 3 Faculty of Veterinary Medicine, Clinic for Ruminants and Swine, Freie Universität Berlin, Berlin, Germany; 4 Department of Biometry, Epidemiology and Information Processing, WHO Collaborating Center for Research and Training for Health at the Human-Animal-Environment Interface, University of Veterinary Medicine, Foundation, Hannover, Germany; 5 Institute for Veterinary Epidemiology and Biostatistics, Freie Universität Berlin, Berlin, Germany; Michigan State University, UNITED STATES

## Abstract

Swellings of the ribs result from severe injury and affected animals are subjected to considerable and prolonged pain and suffering. The knowledge on rib swellings in dairy cows has yet been very limited. Therefore, the present study aimed at determining the prevalence of rib swellings in tie stall housed dairy cows in Germany as well as at identifying associated factors. Mean animal-level prevalence of rib swellings for 2,134 cows was 7.54% with a mean of 7.00% on farm level (range 0.00% - 37.49%). Multivariable mixed logistic regression models including nested random effects were built and factors associated with swellings of the ribs were evaluated for 1,740 dairy cows on 96 farms in Germany. Out of the initial 22 predictors, 8 factors were selected for the final model. Managing dairy cows on a part-time basis (OR 0.49 [CI 0.25–0.98]) appeared to decrease the odds for rib swellings compared with full-time farming. Cattle breeds other than Simmental entailed lower odds for rib swellings (OR 0.29 [CI 0.14–0.59]). Lame cows (OR 2.59 [CI 1.71–3.93]) and cows with wounds and/or swellings of the hocks (OR 2.77 [CI 1.32–5.84]) had more than two times the odds for rib swellings compared with sound animals. The results of the present study can help raising awareness of rib swellings in dairy cows and contribute to the body of evidence on this condition.

## Introduction

Animal welfare has become increasingly important for consumers of milk and beef [1–3] as well as for political decision making [4]. Regulatory framework contributes to the improvement of animal well-being but policy makers need objective parameters to evaluate different housing systems and to set political incentives for improving livestock production. The Animal Care Reference Manual of the National Milk Producers Federation [5] defines animal welfare as being “[…] healthy, comfortable, well nourished, safe, able to express innate behaviour and not suffering from unpleasant states such as pain, fear and distress.” Von Keyserlingk et al. [6] have defined animal welfare as a positive affective state, where animals are able to establish their physiological functions and have the capacity to express natural behavioural patterns. Health disorders that entail pain and suffering are yet in sharp contrast to these aspects [6–8]. Council Directive 98/58/EC in Europe [4] article 3 underscores the specific responsibility of animal owners or keepers to ensure physical integrity of their animals and to minimise avoidable pain, suffering or injury. For the evaluation of animal welfare aspects in different housing systems of dairy cattle, housing-associated traumatic lesions and alterations of the integument as well as lameness have been considered critical in regard to dairy cow comfort [9–11].

Even though an abundance of studies has been presented on traumatic lesions associated with housing conditions [10,12,13] rib swellings in dairy cows have yet received little scientific interest and knowledge is limited [14]. Blowey and Bell [15] have presumed an association between the presence of claw lesions, lameness and swellings of the ribs. Lame cows have difficulties with physiological locomotion and may therefore be prone to falling down. Additionally, lame cows lay down and rise in an unphysiological manner and thus may collide with elements of the stalls [16]. Rib swellings may also be attributable to injuries by the horns of herd mates, transport or mounting by other cows [17]. Paton [18] and Blowey [16] have assumed an association of rib swellings with a low body condition score (BCS) which becomes especially perceivable in the area caudal of the olecranon, at the costochondral junction. However, the mid shaft point of the rib can be also affected, the changes often appear bilaterally and consist of a hard bony enlargement [16]. Knowledge on the prevalence of rib swellings in dairy cows as well as on potential risk factors is very limited. Therefore, the aims of this study were (1) to determine the prevalence of rib swellings in tie stall housed dairy cows and (2) to identify potential risk factors by means of multivariable mixed logistic regression models. Factors associated with management practises, housing conditions, and the individual animal were considered. Knowledge of these factors is of special interest in order to understand relevant on-farm patterns of management and housing that may be associated with an increased prevalence of rib swellings and to work towards a continuous improvement of animal welfare.

## Material and methods

### Herd selection

This study was part of a cross-sectional study on animal health, biosecurity, and housing conditions in German dairy farms. Tie stall farms in three main dairying regions in the North (region 1), East (region 2), and South (region 3) of Germany were included. Study selection and sampling procedures are elaborated on [19–21].

Briefly, farms were randomly sampled for each study region (North: federal states of Lower Saxony and Schleswig-Holstein; East: federal states of Saxony-Anhalt, Brandenburg, Mecklenburg-Western Pomerania, and Thuringia; South: federal state of Bavaria) and stratified by administrative district and farm size. The national animal information database (HIT) as well as farm data from the Milchprüfring Bayern e.V. provided information on farm size within administrative district and study region for sampling. Farms were randomly drawn from these data bases stratified by administrative district and farm size (number of cows) within the federal state and study region. Different scenarios were calculated given a power of 80% and a level of significance of 5% in order to calculate an optimal and feasible sample size. Given these scenarios and considering feasibility, 250 farms were determined to be visited per study region. A response rate of 30–40% was expected. Within each study region, a total amount of 1,250 farms, i.e. 5 times more farms than required for the study, were drawn from the underlying population in order to cover a response rate of at least 20%. Region-specific farm size cut-off values were determined in order to obtain a realistic distribution of farm sizes within the study population and due to structural differences in dairy farming in Germany [22]. Overall, the response rate was far lower than originally expected and overall ranged from 6% to 9% (North: 9%; East: 9%; South: 6%), 253 farms were from region North, 252 from East and 260 from South.

Farms received information on the study and an invitation to participate by mail and chose voluntarily to participate. Every farm was visited once.

Observers (n = 16) were jointly trained during a seminar at the start of the study: Questionnaires and data entry forms were evaluated and discussed, video assessments and live scorings were included. During a subsequent three-month pilot phase observers were trained in farm visits, possible pitfalls were identified and refinements were made. As a result, standard operating procedures could be established.

On-farm data collection was carried out between December 2016 and August 2019. The anonymity of the participating farms was guaranteed in alignment with the German and European data protection legislation.

### On-farm assessments

All lactating and dry animals housed in tie stalls at the day of the farm visit underwent individual scoring for body condition, lameness, rib swellings and changes of the hock, neck, back and tail. Live stall lameness scoring was performed to document weight shifting between feet, sparing one foot while standing, unequal weight bearing when being induced to step from side to side, and standing on the edge of the kerb [23]. Each cow was observed from the rear as well as from a caudolateral perspective during a 90 seconds period of time. If two out of the aforementioned four indicators could be recorded, a cow was classified as lame [23,24]. Body condition scoring was carried out according to a five point scale ranging from 1–5 with 0.25 increment intervals established by Edmonson et al. [25], modified by Metzner et al. [26] for Simmental cattle. BCS was categorised into *underconditioned*, *optimally conditioned*, and *overconditioned* in alignment with what has been presented for different breeds and stages of lactation [27–31]. Elaborations are presented in (S1 File).

**Fig 1:**
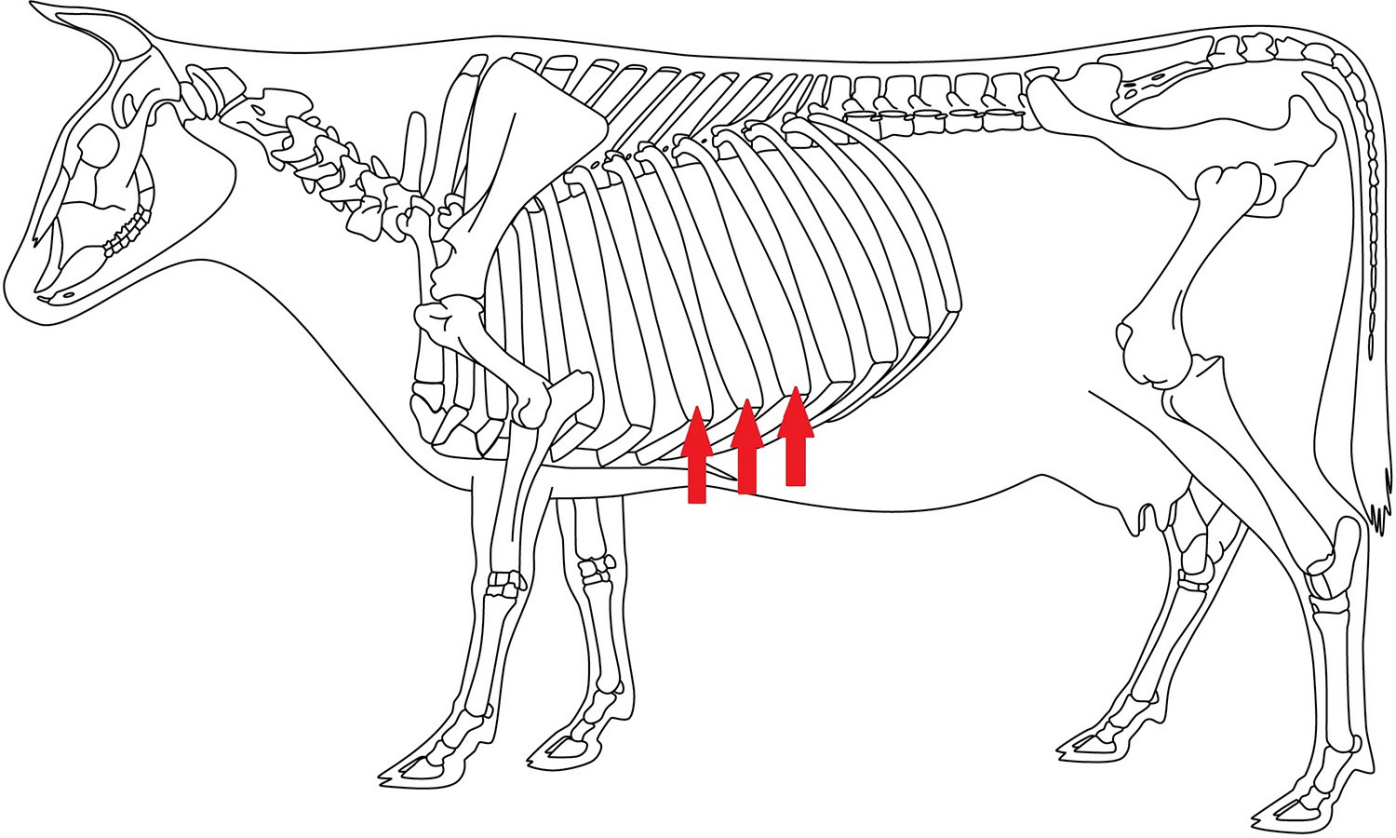
Common predilection site of rib swellings in cattle: Costochondral junctions at 7^th^ - 9^th^ rib.

The presence of rib swellings was visually recorded in the lateral thoracic region between the 7^th^ and the 9^th^ rib at the transition from the bony part to the cartilaginous part of the rib (Fig 1) [32]. Cows were assessed from a caudolateral position.

Skin changes of the hocks were documented using the approach by Regula et al. [33] and Kielland et al. [34]. Accordingly, both tarsal regions were observed from a caudolateral perspective: 1 = no skin change, 2 = hairless patch (visible loss of hair as well as visible hair breakage), 3 = swelling (no wound), 4 = wound (no swelling but any form of disconnection/injury/laceration of the skin), 5 = wound and swelling. A score of 6 (no assessment possible due to solid plaque of manure) was introduced to reflect hocks which were too dirty to be assessed. The most severe skin change present at a time was documented. Strict standard operating procedures were developed at the beginning of the study and during the pilot phase.

Skin changes in the neck area were assessed between the first cervical and the first thoracic vertebra using a score according to Kielland et al. [35]: 1 = no skin change, 2 = hairless patch, 3 = wound and/or swelling. A similar score was applied to record abnormalities of the back in the region between the first cervical and the first caudal vertebra in an area of 10 centimetres on both sides of the median line of the back. Abnormalities of the tail were documented as follows: 1 = no change, 2 = bulge or deviation of the tail, 3 = amputated tail.

Stanchions were selected according to a systematic random sampling and measured for length and width. The median value per farm was calculated. The assumptions of a 95% confidence interval (CI), a standard deviation (SD) of 10, a precision of ± 5 centimeters and a potential number of 10 up to 1,500 stalls on farm were taken as a basis for the following procedure. The number of stanchions on each farm that were to be measured was in accordance with an a priori determined calculation: if up to 29 stanchions with cows were present, 10 were measured, if 30–49 stanchions were present, 15 were measured, and if 50–99 stanchions were present, 17 were measured. In case more than 100 stanchions were present, 18 were measured. Which stanchion needed measuring was counted starting with the first stall. For example, if 30 stanchions were present on a specific farm, every second was due to measuring.

The assessment of slipperiness, stall base (rubber, concrete), gutter design, type of tying system and bedding was conducted in a similar manner to the measurements of stanchions see above.

Slipperiness was assessed as follows: the observers tried to glide on the floor with their boots to ascertain the extent of resistance [36]: 1 = low slipperiness, 2 = moderate slipperiness, 3 = high slipperiness.

Information on the operational type of the farm (main/ supplementary source of income, organic/ conventional farming) and if pasture access or an additional outdoor exercise area were present for cows, was collected during an interview with the farm manager on each farm. Sideline farmers declared that they primarily did a job other than dairy farming and that dairy farming was solely a supplementary source of income.

HIT as well as the national milk recording system (DHI) were used to retrieve data on parity, age, breed, and days in milk.

Data were recorded via questionnaires and data entry forms and manually introduced to a central SQL database immediately after every on-farm assessment. From this database, Microsoft Excel data sheets could be exported for further analyses.

### Data editing and statistical analysis

All statistical analyses were conducted with the R software for statistical computing version 1.2.5033 (R Core Team, 2019) using the R Studio interface. The following packages were implemented: plyr [37], gdata [38], tidyverse [39], tidyr [40], ggstatsplot [41], lme4 [42], lmerTest [43], sjPlot [44], effects [45,46], optimx [47], performance [44], and caret [48].

Statistical unit was the cow. Each predictor underwent descriptive analysis in order to assess the distribution. Univariable analyses were performed on animal level for each factor in regard to the target variable *rib swelling* (1/0) using binary logistic regression. The variable *breed* was sorted into two categories (German Simmental vs. Other) for the multivariable regression. Similarly, the variables *length* and *width of stanchions* were transformed into categorical variables with three categories depending on their distribution and the values of their quartiles (length: < 157.0 centimeters = short, ≥ 157.0 centimeters–<175.0 centimeters = medium, ≥ 175.0 centimeters = high; width: < 99.0 centimeters = narrow, ≥ 99.0 centimeters–<104.0 centimeters = medium, ≥ 104.0 centimeters = large). Similarly, *farm size* was categorised into small (< 22 animals), medium (22–38 animals) and large (≥ 39 animals) based on the number of cows housed on farm.

For the multivariable modelling procedure, lesions of the neck, the back, and the tail were dichotomised (no skin change present vs. observable skin change present). We proceeded similarly for hock lesions: Hocks which scored “6” were from further analysis excluded. Subsequently, the remaining hock scores were sorted into the categories 1 (no skin change observed), 2 (hairless area), and 3 (wound and/or observable swelling).

For the multivariable modelling procedure, a complete cases data set including all potential variables of interest was created. In a manual stepwise forward selection process, multivariable mixed logistic regression models including a nested random effect (*farm* nested within *region*) were created using the glmer function in R. The random effect accounts for random variability within the data as a result of actual differences between farms (within regions), e.g. housing conditions, management practices, and further farm-specific elements across the different dairy operations. In doing so, we payed attention to the fact that effects may differ across farms since animals are subjected to varying on-farm settings. Moreover the random effect also accounts for the clustering of animals within herd.

*Farm size* (categorised) and *season* (*spring*: March, April, May; *summer*: June, July, August, *autumn*: September, October, November; *winter*: December, January, February) were included as fixed effects.

The Akaike’s Information Criterion (AIC) and conditional R^2^ were used to assess the model’s quality after every newly introduced variable. A p-value of ≤ 0.05 was regarded as statistically significant. The model’s quality improved, if a statistically significant decrease of the AIC was detected. The lowest AIC represented the most appropriate model [49]. Furthermore, the R function compare_performance (model 1, model 2, rank = TRUE) from the performance package [44] was implemented. This function allows to rank model based on several criteria. Additionally to the AIC, the BIC, which is more strict in regard to model complexity, as well as conditional R^2^, Intraclass correlation coefficient, and sigma are displayed. We used a combination of these criteria to assess models. Moreover, a Hosmer-Lemeshow test [50] using the performance_hosmer() function from the R package performance was conducted. A p-value of 0.418 indicated the appropriateness of the model. A receiver operating characteristic curve (ROC-curve) was generated (S1 File) using the R package ROCR [51] and the area under the curve (AUC) was calculated to be 0.836 (95% Confidence Interval 0.802–0.870) via the pROC package [52].

The R function vif() from the caret package [48] was applied to control for variance inflation (VIF) and to identify (multi-)collinearity among the predictors. As none of the VIF scores was higher than 5, (multi-)collinearity was determined not to be present [53]. If an interaction between predictors appeared plausible from a biological point of view, this interaction was integrated within the model and the AIC was assessed if a significant improvement was yielded.

## Results

A total number of 2,134 dairy cows on 97 farms in Germany were included in the initial data set of this study, 307 of them in 12 farms from region *North*, 98 in 6 farms from region *East* and 1,729 in 79 farms from region *South*.

The mean animal–level prevalence of rib swellings among these cows was 7.54%. On farm level, the mean prevalence of rib swellings was 7.00% (range 0.00% - 37.49%). Ribs swellings were not present on 39 out of 97 farms (40.21%). On average, farms managed 22 cows and the farm size ranged from one to 108 cows. Descriptive statistics of all variables within the data set are presented in Tables 1 and 2.

**Table 1:** Descriptive statistics of all categorical variables within the initial data set.

Predictor	Categories	n _cows_ (%)	n_cows_ (%) Region *North*	n_cows_ (%) Region *East*	n_cows_ (%) Region *South*
Rib swellings	No Yes	1,973 (92.46) 161 (7.54)	304 (99.02) 3 (0.98)	96 (97.96) 2 (1.04)	1,573 (90.98) 156 (9.02)
BCS	Optimally conditioned Overconditioned Underconditioned	1,333 (65.44) 262 (12.86) 442 (21.70)	154 (57.46) 39 (14.55) 75 (27.99)	37 (45.68) 17 (20.99) 27 (33.33)	1,142 (67.65) 206 (12.20) 340 (20.14)
Breed	Brown Swiss German Holstein German Simmental Other	191 (8.95) 368 (17.24) 1,429 (66.96) 146 (6.84)	1 (0.33) 238 (77.52) 21 (6.84) 47 (15.31)	2 (2.04) 69 (70.41) 10 (10.20) 17 (17.35)	188 (10.87) 61 (3.53) 1,398 (80.86) 82 (4.74)
Back changes	No skin change skin change	2,058 (96.44) 76 (3.56)	302 (98.37) 5 (1.63)	88 (89.80) 10 (10.20)	1,668 (96.47) 61 (3.53)
Hock changes	No skin change Hairless spot Wound and/or swelling	336 (18.14) 1,109 (59.88) 407 (21.98)	90 (31.36) 149 (51.92) 48 (16.72)	20 (22.22) 52 (57.78) 18 (20.00)	226 (15.32) 908 (61.56) 341 (23.12)
Neck changes	No skin change Hairless spot Wound and/or swelling	1,279 (59.54) 724 (33.99) 138 (6.47)	195 (63.73) 97 (31.70) 14 (4.58)	70 (71.43) 23 (23.47) 5 (5.10)	1,005 (58.13) 605 (34.99) 119 (6.88)
Tail changes	No alteration Signs of fracture^1^/ Amputation	1,996 (93.62) 136 (6.38)	279 (90.88) 28 (9.12)	76 (77.55) 22 (22.45)	1,641 (95.02) 86 (4.98)
Lameness	Not Lame Lame	1.673 (78.40) 461 (21.60)	274 (89.25) 33 (10.75)	73 (74.49) 25 (25.51)	1,326 (76.69) 403 (23.31)
Farming on regular / sideline basis	1 (regular) 2 (sideline basis)	1.537 /72.95) 570 (27.05)	294 (95.77) 13 (4.23)	98 (100.00) 0 (0.00)	1,145 (67.27) 557 (32.73)
Farming type	Conventional farming Organic farming	1,915 (89.74) 219 (10.26)	307 (100.00) 0 (0.00)	91 (92.86) 7 (7.14)	1,517 (87.74) 212 (12.26)
Gutter design	Concrete or gutter without grate Gutter with grate	595 (27.89) 1.538 (72.11)	172 (56.03) 135 (43.97)	64 (65.31) 34 (34.69)	359 (20.78) 1,369 (79.22)
Stanchion flooring	Concrete Rubber	488 (23.00) 1,634 (77.00)	133 (43.32) 174 (56.68)	12 (12.24) 86 (87.76)	343 (19.98) 1,374 (79.22)
Pasture access	Yes No	1,000 (46.86) 1,134 (53.14)	307 (100.00) 0 (0.00)	49 (50.00) 49 (50.00)	644 (37.25) 1,085 (62.75)
Exercise	No access to exercise Exercise	1,853 (86.83) 281 (13.17)	255 (83.85) 52 (16.15)	41 (41.84) 57 (58.16)	1,557 (90.05) 172 (9.95)
Presence of bedding material	No bedding material/ low amount Bedding material present	1,977 (92.69) 156 (7.31)	151 (49.19) 156 (50.81)	98 (100.00) 0 (0.00)	1,728 (100.00) 0 (0.00)
Slipperiness	high slipperiness moderately slipperiness low slipperiness	259 (12.14) 1,174 (55.04) 700 (32.82)	33 (10.75) 149 (48.53) 125 (40.72)	13 (13.27) 73 (74.49) 12 (12.24)	213 (12.33) 952 (55.09) 563 (32.58)
Tying system	Grabner tie^2^ Vertical neck frame Collar and chain Other	1,187 (55.65) 330 (15.47) 294 (13.78) 322 (15.10)	187 (60.91) 53 (17.26) 0 (0.00) 67 (21.82)	56 (57.14) 21 (21.43) 5 (5.10) 16 (16.33)	944 (54.63) 256 (14.81) 289 (16.72) 239 (13.83)
Parity	first second ≥third	784 (36.74) 522 (24.46) 828 (38.80)	119 (38.76) 86 (28.01) 102 (33.22)	50 (51.02) 14 (14.29) 34 (34.69)	615 (33.57) 422 (24.41) 692 (40.02)
Season	Autumn Spring Summer Winter	406 (19.03) 817 (38.28) 500 (23.43) 411 (19.26)	48 (15.64) 189 (61.56) 31 (10.10) 39 (12.70)	0 (0.00) 41 (41.84) 57 (58.16) 0 (0.00)	358 (20.71) 587 (33.95) 412 (23.83) 372 (21.52)
Farm-size^3^	< 22 22–39 ≥ 40	521 (24.41) 1,061 (49.72) 552 (25.87)	68 (22.15) 90 (29.32) 149 (48.53)	62 (63.27) 36 (36.73) 0 (0.00)	391 (22.61) 935 (54.08) 403 (23.31)
Observer	1 2 3 4 5 6 7 8 9 10 11 12 13 14 15 16	109 (5.11) 42 (1.97) 13 (0.61) 54 (2.53) 5 (0.23) 34 (1.59) 182 (8.53) 428 (20.06) 213 (9.98) 156 (7.31) 421 (19.73) 68 (3.19) 205 (9.61) 23 (1.08) 36 (1.67) 145 (6.79)	109 42 13 54 0 0 0 0 0 0 0 68 0 0 0 21	0 0 0 0 5 34 0 0 0 0 0 0 0 23 36 0	0 0 0 0 0 0 182 428 213 156 421 0 205 0 0 124

^1^ signs of fracture include the presence of a bulge and a deviation of the tail.

^2^ chain/belt fixed vertically with attached sliding frame around the cow’s neck.

^3^ number of dairy cows equates to farm size.

**Table 2:** Distribution of continuous variables within the initial data set.

Predictor	Mean	Range	1^st^ Quartile	Median	3^rd^ Quartile	n_cows_
Length of stanchion (in centimeters)	169.30	135.50–240.50	157.00	169.30	175.00	2,134
Width of stanchion (in centimeters)	102.40	91.00–131.50	99.00	101.00	102.40	2,134
Days in milk	209.90	0.00–1,112.00	89.00	190.00	303.50	2,099

The results of the univariable analyses are displayed in (S2 File). Out of 23 factors, 15 showed a significant association with the presence of rib swellings.

Omitting missing values, the complete cases data set for the multivariable mixed logistic regression analysis consisted of 1,740 dairy cows on 96 farms. The results of the final model are displayed in Table 3.

**Table 3:** Final multivariable mixed logistic regression model for factors associated with rib swellings in 1,740 dairy cows on 96 farms.

			*Rib Swelling*		
Predictor	Category	Parameter estimate	Odds Ratio	Confidence interval (95%)	P-value
Intercept	-		0.08	0.03–0.24	**<0.001**
BCS					0.071
	Optimal	Reference	-	-	-
	Overconditioned	-0.53	0.59	0.29–1.19	0.140
	Underconditioned	0.35	1.42	0.89–2.27	0.140
Breed					**<0.001**
	German Simmental	Reference	-	-	**-**
	Other	-1.23	0.29	0.14–0.59	**0.001**
Pasture					0.089
	No	Reference	-	-	-
	Yes	-0.53	0.59	0.32–1.08	0.089
Hock changes					**<0.001**
	No skin changes	Reference	-	-	**-**
	Hairless patch	0.23	1.26	0.62–2.54	0.525
	Swelling and/or wound	1.02	2.77	1.32–5.84	**0.007**
Farming on regular / sideline basis					**0.044**
	Regular basis	Reference	-	-	-
	Sideline basis	-0.70	0.49	0.25–0.98	**0.044**
Lameness					**<0.001**
	No	Reference	-	-	-
	Yes	0.95	2.59	1.71–3.93	**<0.001**
Season					0.50
	Autumn	Reference	-	-	-
	Spring	-0.46	0.63	0.31–1.29	0.209
	Summer	-0.37	0.59	0.31–1.57	0.379
	Winter	-0.58	0.56	0.23–1.34	0.194
Farm size					0.40
	< 22 cows	Reference	-	-	-
	22–38 cows	0.10	1.11	0.55–2.22	0.779
	> 39 cows	-0.46	0.63	0.26–1.54	0.314

Out of the initial 22 predictors, 6 factors associated with housing conditions and the individual animal as well as both fixed effects for *season* and *farm size* were maintained within the final model.

The percentage of heterogeneity, i.e. the value of τ for *farm* nested within *region* describing the variance across different farms within the three study regions, was 0.34 in the final model with 0.14 being attributable to *farm*. The total variance of our model was 3.29. Other than Simmental breed (e.g. Brown Swiss, Holstein or others) was found to have lower odds (OR 0.29 [CI 0.14–0.59, p = 0.001) for rib swellings than Simmental cows. Managing on a sideline basis and if dairy farming only provided a supplementary source of income entailed lower odds of rib swellings in dairy cows in the final model (OR 0.49 [CI 0.25–0.98], p = 0.044). Lame cows experienced more than two times the odds of rib swellings compared with non-lame animals (OR 2.59 [CI 1.71–3.93], p < 0.001). As for hock changes, the presence of a wound and/or a swelling entailed higher odds for the presence of ribs swellings in the individual animal (OR 2.77 [CI 1.32–5.84], p = 0.007). This association was statistically significant compared with animals without skin changes in the tarsal area.

## Discussion

The aim of the present study was to assess the prevalence of rib swellings as a welfare factor in dairy cows housed in tie stall facilities in Germany and to evaluate potential risk factors. This is of particular interest as swellings of the ribs are likely to be a consequence of severe injury and hence are associated with considerable, mostly prolonged pain and suffering in affected animals [54,55].

In addition to rib swellings there were other animal welfare factors taken into account.

Lameness is regarded as one of the most important matter for animal welfare in dairy production [56–58]. Hock lesions can be very painful and on their part cause lameness [59]. Lameness, and therefore hock lesions, have considerable adverse effects on milk yield, reproductive performance, life expectancy and general well-being [60,61] so an association among mutual factors can be assumed.

Even though an abundance of studies has evaluated indicators of animal welfare in modern production facilities, rib swellings in dairy cows have yet received little scientific attention, potentially due to the fact that they are mostly chronic lesions when they are discovered which are not in a particular focus by both veterinarians and farmers and usually do not experience veterinary treatment [16]. To our knowledge, the present study is the first work to determine the prevalence of rib swellings in tied dairy cows and to evaluate potential risk factors.

As early as 2012, Merle et al. [22] described structural differences of farming within Germany. To meet this requirement we included the three regions in our study. Tie stall housing in Germany is similar to other countries in Europe, e.g. Switzerland and Austria. So we could assume that the results are readily applicable to other tie stall settings.

The variance of our model is 3.29. Structural differences between farming practises and management exist in the dairy sector throughout Germany [22].: For example, larger dairies are present in East Germany (region East of this study) compared with other parts of the country. Another characteristic to be aware of is the integration of pasture access in dairy production which is common in North Germany (region North) as well as in parts of Bavaria (region South) while largely absent in East Germany. The average farm size is larger in the Northwest than in other regions and soil fertility within Germany varies greatly. These and other differences may be the source of the 20% variance of *region*. This may be an explanation for the range in mean farm-level prevalence of rib swellings and underscores the importance of individual, farm-specific strategies to evaluate the situation and to develop control programmes. This is supported by Boyling [62] who similarly reported a fairly marked range of farm-level prevalences between 3.5% and 26.8% with a mean of 9.70% which is similar to the present study but in free stall barns. Apart from this reasoning, small farms with a small amount of cows may tend to reach high or low farm prevalence with only a few animals being housed. Hence, the impact of one affected animal on the farm-specific prevalence of rib swellings is more pronounced than in larger farms.

A study of Blowey, Bell and Boyling [32] mentions a mean prevalence of rib swellings of 14.7% in 1,998 dairy cows in 13 herds housed in cubicle systems with different bedding types. Witchell [55] found 16.1% of dairy cows in cubicle systems to be affected. As for the current study, it is important to be aware, that solely the presence of rib swellings was assessed. Hence, no assumption can be made about the underlying pathology, e. g. trauma-associated swellings, abscesses, fractures and others.

Enrollment in this study was on a voluntary basis. Farmer characteristics play a pivotal role in regard to animal welfare and to the way they manage their farms as well as to how they communicate and interact with veterinary consulting [63–66]. Proactive farmers are more open to external advice and particularly interested in preventive rather than reactive approaches of tackle issues of animal welfare and health on their farms [67–69]. This may have rendered these farmers more intrinsically motivated to participate in the present study whereas famers with a different mindset may not have had this level of motivation to be enrolled.

Even with a low number of scorers, consistent and reliable records e.g. for locomotion are difficult to obtain and inter-observer reliability values often are unable to obtain the expected levels. Therefore, animals may be misclassified in regard to the outcome of interest and relevant associations may be covered up during modelling or spurious associations may arise [70–73]. In regard to locomotion scores, a very recent study has underscored the importance of including observer within the modelling procedure in order to draw reliable inference [20]. In the current study, assessment of inter-observer reliability in regard to rib swellings was not possible. Therefore, observer was included as a fixed effect throughout the modelling process in the current study in order to account for potential observer effects. However, it is important to be aware that a high number of observers was included and some observers had a very low number of observations. This not only rendered the models unable to converge but also could not yield estimates for single observers. Therefore, the effect of observer was solely modelled in a univariable context. From this, we can say that while for some observers, estimates could not be produced, some observers did not appear to have a relevant association in regard to rib swellings and relevant associations were present for single observers. When looking at the results of the present work, this needs to be taken into account to avoid misinterpretation and to be aware of potential limitations. For future studies in this context, we recommend assessing inter-observer reliability.Lameness in the present study was assessed using the SLS [23,24]. It is important to be aware of the fact that a moderate sensitivity of 0.54–0.77 was determined by Leach et al [23] compared with the Sprecher locomotion scoring system [74]. Hence, lameness may be underestimated on average by 27% (11–37%) when using the SLS approach [23]. As a consequence, the true lameness prevalence may well be higher among tied dairy cows than reported in the present work. Jewell et al. [12] also used the SLS in their study and reported 15.3% (95% CI 12.5–18.6) of cows to be lame with a farm-level prevalence of lameness of 0–30.6%. In a study by Bouffard et al. [75] 25% of cows scored for lameness using the SLS approach were identified as lame. Even though lameness may have been underestimated because of the method of assessment, the prevalence reported in the current work is in accordance with the extant literature.

Furthermore, we are convinced to have attained a high level of standardisation due to an intensive study protocol including standard operation procedures from enrollment to data collection, plausibility checks, and analysis. Moreover, the wide variation between herds in regard to the prevalence of rib swellings as well as the aforementioned results from previous studies supports the outcomes of the present study.

Our model showed a significant relationship between the occurrence of rib swellings and lameness. The causality yet is still not clear and needs further investigations. Even moderately lame cows extend their lying duration about approximately 45 minutes [76] which may foster the development of decubital areas in the costochondral area of the ribs. Relatively more rib swellings were present in stanchions covered with rubber mats compared with stalls where bare concrete was the type of stall base. However, rubber mats are commonly covered with only sparse amounts of bedding which may explain the increased occurrence of rib swellings. This setting of bedding amount and type of stall base clearly requires deeper investigations. Moreover, the way to lie down is unphysiological in lame cows. Lame cows may be more prone to either slipping and falling down abruptly or to harmful collisions with elements of stall design, especially in short stalls that are common in tie stall facilities [8,76] which can result in an increased occurrence of rib swellings [16].

Changes of the hocks increased the odds for rib swellings (OR 2.77 [CI 1.32–5.84]) in our final multivariable mixed logistic regression model. Hock injuries also seem to be associated with lameness [77]. Witchell [54] described a higher risk of hock injuries in lame cows, potentially through an increasing lying time that means an expansion of contact time between the lying surface and the hocks [78]. Similar results have been presented by a recent study on lameness in tie stall facilities by Oehm et al. [77]. Swellings and/or wounds on the hocks increased the odds of lameness according to them by 2.57. These two variables appear to be closely associated and may share a common risk setting. Accordingly, lameness and hock lesions may have a similar effect on the occurrence of rib swellings. Hence, even though hock lesions (and lameness) may be causally involved in the occurrence of rib swellings, they are a common finding in dairy cows and simply may therefore be associated with the condition.

Simmental cows seem to have a higher risk to get rib swellings than other breeds like Brown Swiss, German Holstein and others. As studies on Simmental cows in particular are limited compared with studies on Holstein cows, it is difficult to provide an evidence-based explanation for this associations.

Part-time-farmers had lower odds of rib swellings on their farm compared with farmers where dairy farming provided the main source of income. Because of a lack of literature for farming in sideline basis we can only speculate about the reasons. One point can be an association with farm size, although farm size itself shows no significant association in our final model but a significant association in univariable analysis with part and full time farming.

## Conclusions

Swellings of the ribs tend to be common on some dairy farms housing their cows in tie stalls whereas other farms do not experience the problem. Knowledge on the occurrence of rib swellings in dairy cows has been scarce. The results of the present study suggest that their occurrence is closely associated with a setting of animal-related and farm-specific factors like hock lesions, breed and lameness.

## Supporting information

S1 File BCS categories. (PDF)

S2 File Results of the univariable analyses of all factors with the target variable rib swelling. (PDF)

S3 File ROC-curve. (PDF)

S4 File Interview guide in English. (PDF)

S5 File Interview guide in the original language German. (PDF)

S6 File Directed acyclic graph. (PDF)

S1 Dataset (XLSX)
