# Supplementary material for: Prevalence of and factors associated with swellings of the ribs in tie stall housed dairy cows in Germany
Source: PLoS One. 2022 Jul 15;17(7):e0269726. doi: 10.1371/journal.pone.0269726 (PMC9286234; doi:10.1371/journal.pone.0269726)
Supplement: S1 File — (PDF) [file pone.0269726.s001.pdf]

S1 BCS categories

Days in Milk	Breed								
	Holstein			Brown Swiss			Simmental/Other		
	Under	Optimal	Over	Under	Optimal	Over	Under	Optimal	Over
0 - 29	≤ 2.75	3.0 – 3.75	> 3.75	≤ 2.75	3.0 – 3.75	> 3.75	≤ 3.25	3.5 – 4.25	> 4.25
30 – 99	≤ 2.5	2.75 – 3.25	> 3.25	≤ 2.5	2.75 – 3.25	> 3.25	≤ 3.0	3.25 – 4.0	> 4.0
100 - 199	≤ 2.5	2.75 – 3.25	> 3.25	≤ 2.5	2.75 – 3.25	> 3.25	≤ 3.0	3.25 – 4.0	> 4.0
200 – 299	≤ 2.75	3.0 – 3.75	> 3.75	≤ 2.75	3.0 – 3.75	> 3.75	≤ 3.25	3.5 – 4.25	> 4.25
> 300	< 3.25	3.25 – 3.75	> 3.75	< 3.25	3.25 – 3.75	> 3.75	< 3.75	3.75 – 4.25	> 4.25

S1: BCS categories in accordance with stage of lactation and breed (Heuwieser und Mansfeld, 1992; Kritzinger und Schoder, 2009a, 2009b; Kritzinger et al., 2009; Martin et al., 2014).
