# Supplementary material for: Prevalence of and factors associated with swellings of the ribs in tie stall housed dairy cows in Germany
Source: PLoS One. 2022 Jul 15;17(7):e0269726. doi: 10.1371/journal.pone.0269726 (PMC9286234; doi:10.1371/journal.pone.0269726)
Supplement: S2 File — (PDF) [file pone.0269726.s002.pdf]

S2 Results of the univariable analyses of all factors with the target variable *rib swelling*.

Predictor<br>- Categories	Parameter estimate	Standard error	Odds ratio	Confidence Interval (95 %)	P-value
BCS					**0.011**
- Optimally conditioned	Reference	-	-	-	-
- Overconditioned	-0.60	0.33	0.55	0.27 – 0.99	0.063
- Underconditioned	0.35	0.18	1.41	0.97 – 2.03	0.067
Breed					
- Other	Reference	-	-	-	
- German Simmental	1.21	0.23	3.35	2.16 – 5.43	**<0.001**
Back changes					
- No skin change	Reference	-	-	-	-
- Wound	0.77	0.34	2.15	1.06 – 4.01	**0.023**
Hock changes					**<0.001**
- No skin change	Reference	-	-	-	-
- Hairless spot	0.12	0.28	1.13	0.67 – 2.02	0.663
- Wound and/or swelling	1.16	0.29	3.18	1.86 – 5.73	**<0.001**
Neck changes					**0.004**

- No skin change	Reference	-	-	-	-
- Hairless spot	0.57	0.17	1.76	1.26 – 2.46	**0.001**
- Wound and/or swelling	0.19	0.35	1.21	0.58 – 2.29	0.584
Tail changes[1]					
- No skin changes	Reference	-	-	-	-
- Signs of fracture/Amputation	-0.02	0.34	0.98	0.47 – 1.81	0.945
Lameness					
- Not lame	Reference	-	-	-	-
- lame	1.16	0.17	3.20	2.30 – 4.45	**<0.001**
Farming on regular/sideline basis					
- regular	Reference	-	-	-	-
- sideline	-0.43	0.21	0.65	0.43 – 0.96	**0.037**
Farming type					
- conventional	Reference	-	-	-	-
- organic	-0.58	0.33	0.56	0.27 – 1.02	0.082
Gutter design					
- Concrete or gutter without grate	Reference	-	-	-	-
- Gutter with grate	0.79	0.22	2.21	1.45 – 3.50	**<0.001**

Stanchion flooring					
- Concrete	Reference	-	-	-	-
- Rubber	0.38	0.21	1.46	0.97 – 2.26	0.080
Pasture access					
- No	Reference	-	-	-	-
- Yes	-0.88	0.18	0.41	0.29 – 0.59	**<0.001**
Presence of bedding material					
- None/ low amount	Reference	-	-	-	-
- Bedding material present	-2.61	1.01	0.07	0.00 – 0.33	**0.009**
Slipperiness					0.6
- High	Reference	-	-	-	-
- Moderate	-0.16	0.25	0.85	0.53 – 1.44	0.527
- Low	0.02	0.27	1.02	0.62 – 1.76	0.929
Tying system					**0.005**
- Grabner tie	Reference	-	-	-	-
- Collar and chain	-0.50	0.27	0.61	0.35 – 1.00	0.064
- Other	-0.88	0.30	0.42	0.22 – 0.72	**0.004**
- Vertical neck frame	-0.35	0.24	0.71	0.43 – 1.11	0.152

Parity					**0.018**
- First	Reference	-	-	-	-
- Second	-0.15	0.24	0.86	0.53 – 1.36	0.518
- ≥ Third	0.40	0.19	1.48	1.03 – 2.15	**0.034**
Exercise area present					
- No	Reference	-	-	-	-
- Yes	-0.34	0.27	0.71	0.40 – 1.18	0.210
Season					0.11
- Autumn	Reference	-	-	-	-
- Spring	-0.47	0.23	0.62	0.40 – 0.98	**0.037**
- Summer	-0.17	0.24	0.84	0.53 – 1.35	0.479
- Winter	-0.013	0.24	0.99	0.61 – 1.59	0.956
Farm size					**0.004**
- < 22 cows	Reference	-	-	-	-
- 22 – 38 cows	0.50	0.21	1.64	1.10 – 2.53	**0.019**
- > 39 cows	-0.10	0.26	0.91	0.54 – 1.53	0.716
Observer[2]					**<0.001**
- 8	Reference	-	-	-	**-**

- 1	-	-	0.00	N/A	0.980
- 2	-	-	0.00	N/A	0.987
- 3	-	-	0.00	N/A	0.993
- 4	-0.56	0.75	0.57	0.09 – 1.98	0.454
- 5	-	-	0.00	N/A	0.996
- 6	-0.80	1.03	0.45	0.02 – 2.22	0.440
- 7	0.86	0.29	2.36	1.33 – 4.21	**0.003**
- 9	-0.68	0.43	0.50	0.20 – 1.12	0.114
- 10	0.53	0.33	1.70	0.87 – 3.21	0.109
- 11	0.69	0.25	2.00	1.24 – 3.30	**0.005**
- 12	-	-	0.00	N/A	0.984
- 13	0.53	0.30	1.70	0.92 – 3.07	0.083
- 14	-	-	0.00	N/A	0.991
- 15	-0.86	1.03	0.42	0.02 – 2.09	0.407
- 16	0.20	0.37	1.22	0.57 – 2.46	0.594
Length of stanchions (categorised)[3]					**0.001**
- Short	Reference	-	-	-	**-**
- Medium	-0.25	0.19	0.78	0.54 – 1.12	0.175

- Long	-0.89	0.26	0.41	0.24 – 0.67	**<0.001**
Width of stanchion (categorised)[4]					0.20
- Narrow	Reference	-	-	-	-
- Medium	0.31	0.21	1.37	0.91 – 2.11	0.143
- broad	0.08	0.24	1.08	0.67 – 1.74	0.753
Days in milk (categorised)[5]					0.10
- < 89 days	Reference	-	-	-	-
- 89 – 303.5 days	-0.03	0.19	0.97	0.67 – 1.43	0.876
- > 303.5 days	-0.45	0.25	0.64	0.39 – 1.03	0.068

[1]Since no cow with an amputated tail also had a rib swelling, the model was not able to calculate a value for this predictor

[2] Some observers had a very low number of observations and values were not estimable

[3]length of stanchions was categorized according to the distribution of the measured values (≤ 158.00 cm; > 158.00 cm – 175.00 cm; > 175.00 cm).

[4]width of stanchions was categorized according to the distribution of the measured values (≤ 99.0 cm; 99.0 cm – 104.0 cm; >104.0 cm)

[5] days in milk was categorized according to the distribution of the measured values (89≤ days; 89 – 303.50 days; 303.50 > days)
