# Supplementary figures and images for: Prevalence of and factors associated with swellings of the ribs in tie stall housed dairy cows in Germany
Source: PLoS One. 2022 Jul 15;17(7):e0269726. doi: 10.1371/journal.pone.0269726 (PMC9286234; doi:10.1371/journal.pone.0269726)

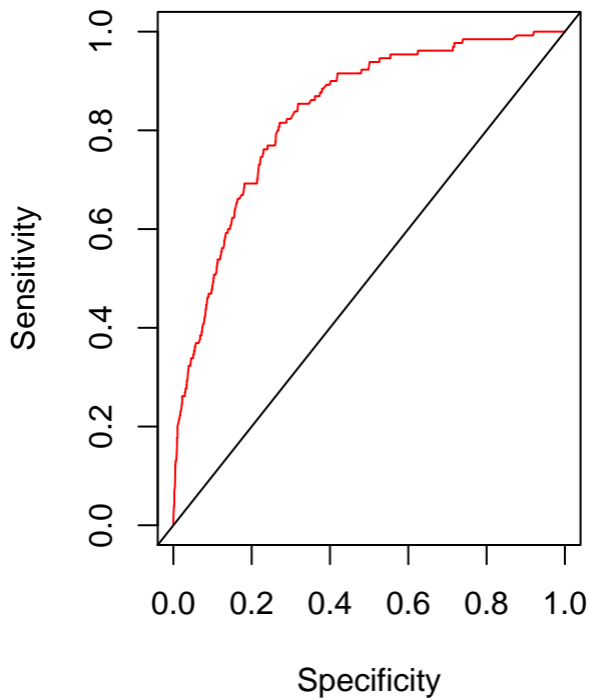

Supplement: S3 File — (PDF) [file pone.0269726.s003.pdf]

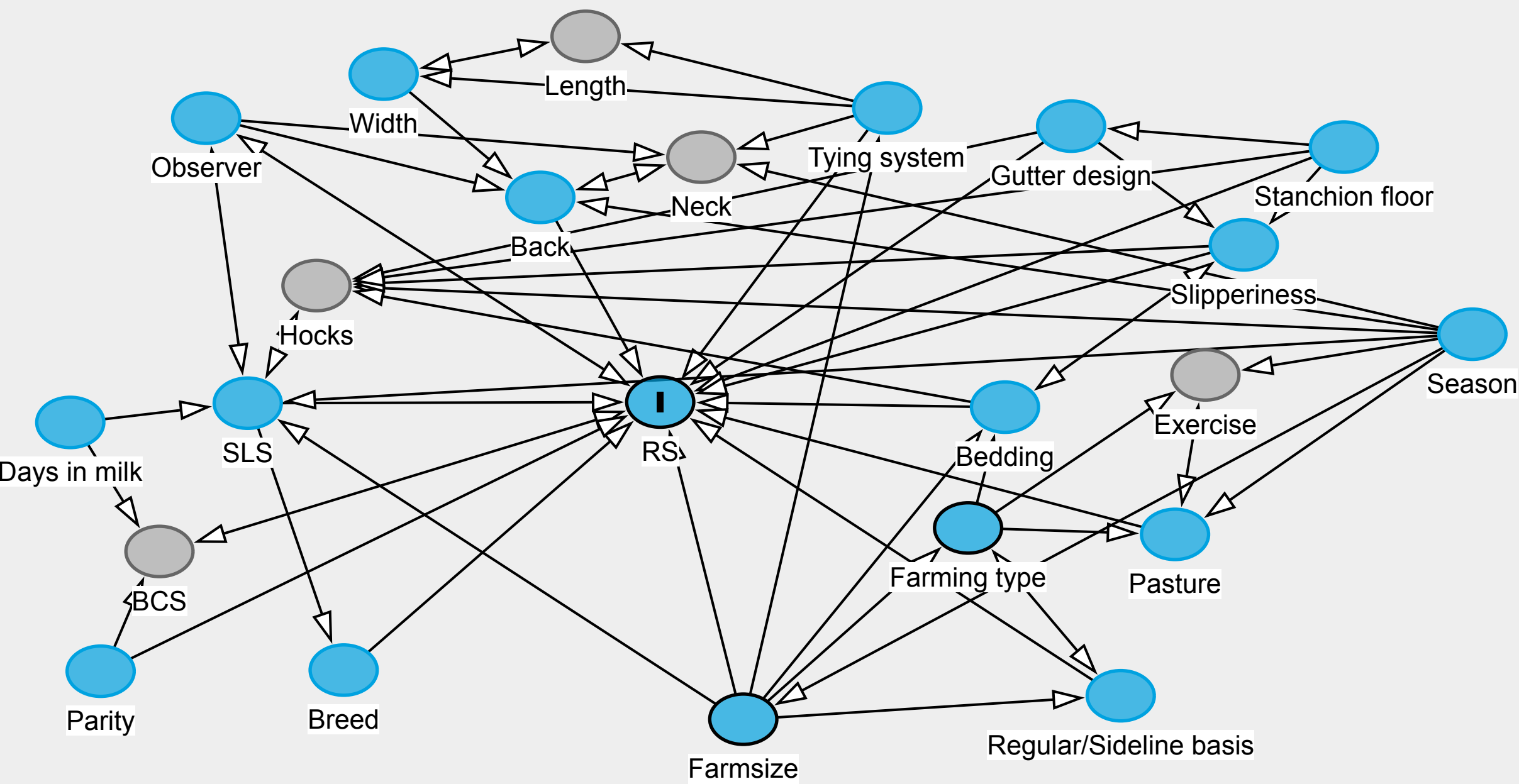

Supplement: S5 File — (PDF) [file pone.0269726.s005.pdf]
