# Supplementary material for: Prevalence of and factors associated with swellings of the ribs in tie stall housed dairy cows in Germany
Source: PLoS One. 2022 Jul 15;17(7):e0269726. doi: 10.1371/journal.pone.0269726 (PMC9286234; doi:10.1371/journal.pone.0269726)
Supplement: S4 File — (PDF) [file pone.0269726.s004.pdf]

Date	Farm ID			
Interviewer	Interviewee			
This questionnaire contains questions about all potential aspects of this farm. The questionnaire is standardised in order to be able to compare farms. therefore, all questions will be read out loud exactly as they are written within the questionnaire. Please answer to them in a short manner. If a question remains unclear, feel free to tell.				
**A. Structure**				
A1. Is your farm the main source of income or a supplementary source of income				
main source	supplementary income	I do not know	not specified	
A2. Is this farm managed conventionally or according to organic farming principles?				
conventional	organic	I do not know	not specified	

Date	Farm ID						
Interviewer							
Interviewee							
A. Pasture							
**A1. Is pasture access provided? If yes, which group of animals is pastured during which season?**							
	young stock	heifers	early lactating cows	cows in mid-lactation	cows in late lactation	early dry cows	cows in late dry period
no							
summer							
year round							
**A2. Is exercise area provided? If yes, which group of animals during which season?**							
	young stock	heifers	early lactating cows	cows in mid-lactation	cows in late lactation	early dry cows	cows in late dry period
no							
summer							
year round							
